# The Vitamin D Receptor and T Cell Function

**DOI:** 10.3389/fimmu.2013.00148

**Published:** 2013-06-18

**Authors:** Martin Kongsbak, Trine B. Levring, Carsten Geisler, Marina Rode von Essen

**Affiliations:** ^1^Department of International Health, Immunology and Microbiology, Faculty of Health and Medical Sciences, University of Copenhagen, Copenhagen, Denmark

**Keywords:** vitamin D receptor, T cell function, vitamin D, signaling, expression, activity

## Abstract

The vitamin D receptor (VDR) is a nuclear, ligand-dependent transcription factor that in complex with hormonally active vitamin D, 1,25(OH)_2_D_3_, regulates the expression of more than 900 genes involved in a wide array of physiological functions. The impact of 1,25(OH)_2_D_3_-VDR signaling on immune function has been the focus of many recent studies as a link between 1,25(OH)_2_D_3_ and susceptibility to various infections and to development of a variety of inflammatory diseases has been suggested. It is also becoming increasingly clear that microbes slow down immune reactivity by dysregulating the VDR ultimately to increase their chance of survival. Immune modulatory therapies that enhance VDR expression and activity are therefore considered in the clinic today to a greater extent. As T cells are of great importance for both protective immunity and development of inflammatory diseases a variety of studies have been engaged investigating the impact of VDR expression in T cells and found that VDR expression and activity plays an important role in both T cell development, differentiation and effector function. In this review we will analyze current knowledge of VDR regulation and function in T cells and discuss its importance for immune activity.

## Introduction

The purpose of the immune system is to recognize and clear pathogens from the body. However, occasionally unwanted immune reactions against self-tissue that lead to autoimmune diseases occur. The frequency of autoimmune diseases such as type 1 diabetes mellitus (Staples et al., 2003; Sloka et al., 2010), rheumatoid arthritis (Vieira et al., 2010), multiple sclerosis (MS) (Hogancamp et al., 1997), and inflammatory bowel disease (Khalili et al., 2012) has been linked to geographic location with a higher incidence of these diseases at higher degrees of latitude. One explanation of this geographical distribution is low exposure to sunlight and hence lower levels of vitamin D (25(OH)D_3_) at higher degrees of latitude, as confirmed by studies showing an association between low serum levels of 25(OH)D_3_ and development of autoimmune diseases (Pierrot-Deseilligny and Souberbielle, 2010; Rossini et al., 2010; Greer et al., 2012). Low serum levels of 25(OH)D_3_ have also been linked to higher susceptibility to infections such as tuberculosis (Nnoaham and Clarke, 2008), influenza (Cannell et al., 2006; Grant, 2008), HIV (Rodriguez et al., 2009), respiratory syncytial virus (Grant, 2008), and viral infections of the upper respiratory tract (Ginde et al., 2009). It is therefore apparent that vitamin D plays a role in immune modulation. A recent acknowledgment that the majority of immune cells expresses the vitamin D receptor (VDR) (Kreutz et al., 1993; Hewison et al., 2003; Baeke et al., 2010; von Essen et al., 2010; Geldmeyer-Hilt et al., 2011; Joseph et al., 2012) and also the enzyme CYP27B1 used for internal conversion of circulating 25(OH)D_3_ to the VDR-ligand 1,25(OH)_2_D_3_ (Hewison et al., 2003; Baeke et al., 2010) has further strengthen this perception.

## Vitamin D, VDR, and T Cell Function

### Mechanism of 1,25(OH)_2_D_3_ action

The cellular actions of 1,25(OH)_2_D_3_ are mediated by the VDR, a ligand-dependent transcription regulator molecule belonging to the superfamily of nuclear receptors. In the absence of 1,25(OH)_2_D_3_-VDR is mainly distributed to the cytoplasm (Nagpal et al., 2005). Interaction of VDR with its ligand 1,25(OH)_2_D_3_ induces formation of two independent protein interaction surfaces on the VDR, one that facilitates association with the retinoid X receptor (RXR) necessary for DNA binding, and one that is required for recruitment of co-regulators necessary for gene modulation (Pike et al., 2012). Following interaction with 1,25(OH)_2_D_3_-VDR dimerizes with RXR and translocates to the nucleus where it binds to vitamin D response elements (VDRE) in vitamin D responsive genes. Depending on the target gene either co-activators or co-repressors are attracted to the VDR/RXR complexes to induce or repress gene transcription (Nagpal et al., 2005; Pike et al., 2012; Haussler et al., 2013). Even though details of how these co-regulatory complexes work are only slowly beginning to emerge, it is now evident that they include ATPase-containing nucleosomal remodeling capabilities, enzymes with chromatin histone modifying abilities (e.g., acetyl- or methyl-transferases) and proteins involved in recruitment of RNA polymerase II (Pike et al., 2012; Haussler et al., 2013). Besides regulation through VDRE, VDR can inhibit genes by antagonizing certain transcription factors (Alroy et al., 1995; Takeuchi et al., 1998; Towers and Freedman, 1998). One such example is VDR-dependent inhibition of the T cell cytokine IL-2. Here, VDR first competes with the transcription factor NFAT1 for binding to the enhancer motif of AP1 and subsequently VDR binds to c-Jun. This co-occupancy of VDR-c-Jun to AP1 leads to inhibition of IL-2 expression. The VDR inhibition of the IL-2 gene requires that VDR dimerizes with RXR, illustrating a need for 1,25(OH)_2_D_3_ (Alroy et al., 1995; Takeuchi et al., 1998; Towers and Freedman, 1998). Overall, the cellular action of vitamin D therefore depends on sufficient production and delivery of 1,25(OH)_2_D_3_ and adequate expression of VDR and its associated proteins. Since the VDR in 1983 was reported to be expressed in immune cells (Bhalla et al., 1983; Provvedini et al., 1983) an increasing effort to elucidate the importance of vitamin D on immune function has been undertaken. It has become increasingly clear that a major mechanism to control the immune regulatory effect of vitamin D is adjustment of the expression level and activity of the VDR.

### VDR expression and development of T cells

Due to the importance of T cells in protective immunity and in development of inflammatory and autoimmune disorders, several studies have examined the impact of VDR expression on T cell development, differentiation, and function. One approach to determine the role of VDR expression in development of T cells has been to study mice lacking the VDR (VDR-KO). These mice show normal numbers of CD4^+^ and CD8^+^ T cells including naturally occurring CD4^+^ FoxP3^+^ regulatory T cells (nTreg) (Yu et al., 2008), suggesting that VDR is not required for development of either of these cell types. A study performed by Hayes and coworkers using a mouse model with defective VDR in only the T cells confirmed that VDR is not essential for development of either conventional CD4^+^ T cells, CD8^+^ T cells, or CD4^+^ FoxP3^+^ nTreg cells (Mayne et al., 2011). Even so, VDR-KO mice appear to have a more vigorous immune response as seen by their increased risk of development of autoimmune diseases (Froicu et al., 2003; Froicu and Cantorna, 2007), and the enhanced response of VDR-KO T cells in mixed lymphocyte reactions (Froicu et al., 2003). In a series of studies Cantorna and coworkers have establish that the increased immune reactivity observed in VDR-KO mice in part is caused by a failure to develop the two regulatory T cell subsets, invariant NKT (iNKT) cells and CD8αα/TCRαβ T cells (Yu and Cantorna, 2008; Yu et al., 2008; Bruce and Cantorna, 2011). iNKT cells are a subset of T cells with a regulatory role in autoimmunity and infection (Godfrey et al., 2000; Bendelac et al., 2001; Singh et al., 2001). CD8αα T cells are mainly present in the gut, where they help maintain tolerance and suppress inflammation by dampening the response to a large number of gut antigens (Poussier et al., 2002; Cheroutre, 2004). The VDR-KO mice have significant fewer iNKT cells, due to a block in development as VDR is implicated in Tbet expression and conversion to the mature NK1.1 expressing mature iNKT cell. The few iNKT cells present in the periphery are furthermore functionally defective (Yu and Cantorna, 2008; Ooi et al., 2012). Like the iNKT cells, there are also fewer CD8αα/TCRαβ precursors in the thymus of VDR-KO animals. Moreover, to complete development CD8αα/TCRαβ cells must travel from the thymus to the gastrointestinal tract where IL-15 induces proliferation and upregulation of CD8αα. Due to decreased levels of IL-15 receptor expression VDR-KO CD8αα/TCRαβ cells proliferate poorly, resulting in a diminished mature CD8αα/TCRαβ population in the VDR-KO gut (Yu et al., 2008; Bruce and Cantorna, 2011; Ooi et al., 2012). These data illustrate that in contrast to conventional T cells, VDR expression is mandatory for development of both iNKT cells and CD8αα/TCRαβ T cells.

### VDR expression and differentiation of T cells

Adaptive immune responses require priming and proliferation of naïve T cells followed by migration of the resulting effector T cells to the site of infection. Antigen-specific triggering of TCRs expressed on the surface of antigen-naïve T cells together with co-stimulation induces intracellular signaling events that promote upregulation of the VDR (Provvedini et al., 1983; von Essen et al., 2010; Joseph et al., 2012). This activation-induced upregulation of VDR in naïve human T cells encourages 1,25(OH)_2_D_3_-VDR signaling. 1,25(OH)_2_D_3_-VDR signaling induces upregulation of the VDRE containing enzyme PLC-γ1, which is a central molecule in the classical TCR signaling pathway. Following VDR-induced PLC-γ1 upregulation classical TCR signaling is established and full T cell activation accomplished (von Essen et al., 2010). VDR expression therefore contributes to priming of naïve human T cells. Interestingly, this VDR-induced PLC-γ1 upregulation is not a mechanism involved in T cell priming of mouse T cells, as naïve mouse T cells already expresses substantial amounts of PLC-γ1 (Ericsson et al., 1996). In order for T cells to proliferate they need the cytokine IL-2. IL-2 is produced and secreted by T cells in response to antigen-induced T cell stimulation. In an autocrine and paracrine fashion IL-2 binds to high affinity IL-2 receptors on the same or adjacent T cells, inducing cell proliferation and hence a clonally expanded population of antigen-specific effector T cells (Cantrell and Smith, 1984; Smith, 1988). As VDR expression has been shown to inhibit transcription of the IL-2 gene (Alroy et al., 1995; Takeuchi et al., 1998), it is likely that upregulation of VDR serves as a negative feedback mechanism to control potential overreactions of the immune system. Besides inducing the early priming phase of naïve human T cells and possibly ensuring immune integrity, Mathieu and coworkers showed that a 1,25(OH)_2_D_3_ agonist drastically changed the surface expression of homing receptors on both CD4 and CD8 T cells, resulting in a profile corresponding to an increased migration ability to sites of infection (Baeke et al., 2011); and hence implying a role for VDR in all phases of T cell differentiation.

In agreement with a suggested role of VDR in preventing immune overreaction, a changed distribution of naïve and antigen-experienced T cells was observed in a VDR-KO study performed by Bruce et al. (2011). The CD4^+^ T cells had a more activated phenotype and readily developed into the proinflammatory Th17 effector cells that produced twice as much IL-17 as their WT counterparts *in vitro* (Bruce et al., 2011). Furthermore, vitamin D has been shown to modify the phenotype of antigen presenting dendritic cells (DC) to a more tolerogenic phenotype that favors differentiation of inducible Treg (iTreg) cells instead of the inflammatory Th1 and Th17 cells (Griffin et al., 2001; Adorini et al., 2003; Adorini and Penna, 2009). In VDR-KO mice, the frequency of total DC populations were not affected, but a significant reduction in tolerogenic DCs was observed (Bruce et al., 2011). In accordance with the reduced population of tolerogenic DCs and increased population of activated inflammatory T cells, a decrease in the population of iTregs that differentiated from naïve T cells was observed (Bruce et al., 2011). This lead to an increased pathogenic potential of the T cell population, which manifested in development of more severe experimental inflammatory bowel disease (Bruce et al., 2011). These observations emphasize the importance of VDR expression in controlling the balance between effector and tolerogenic cells.

### VDR expression and function of T cells

**Figure 1:**
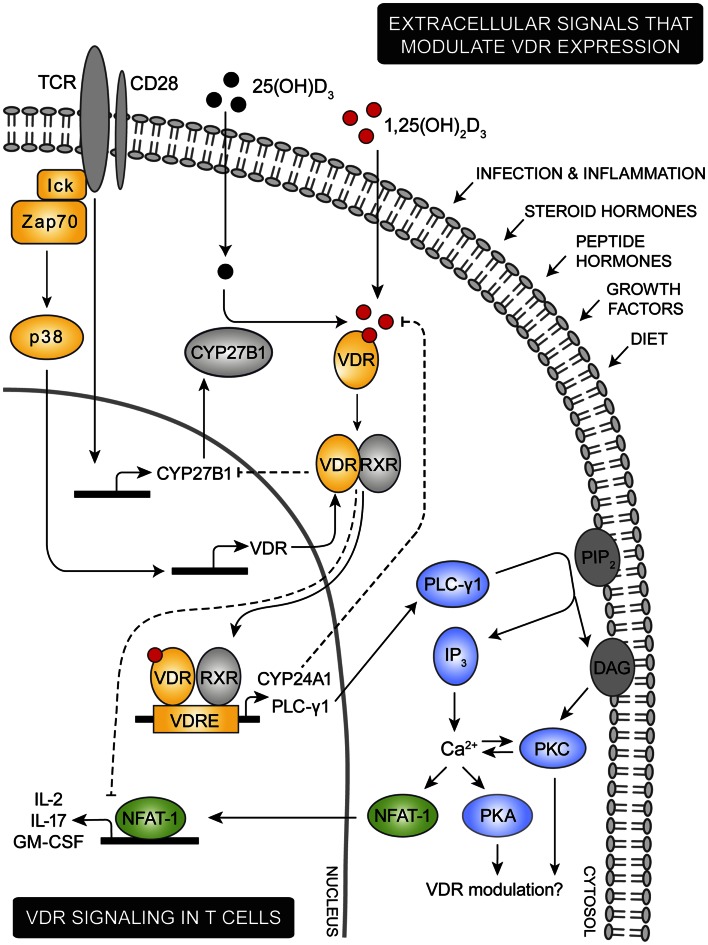
**Proposed model for VDR signaling in T cells**. Various extracellular signals including infection, inflammation, steroid and peptide hormones, and diet are involved in regulation of the intracellular VDR level. During an immune response the TCR is triggered by specific antigens, inducing a cascade of intracellular signaling events. Among these, lck and ZAP-70 are activated leading to activation of the p38 kinase which in naïve human T cells induce expression of VDR. TCR triggering also promotes expression of the 1,25(OH)_2_D_3_ synthesis enzyme CYP27B1. Through intrinsic synthesis of 1,25(OH)_2_D_3_ and uptake of 1,25(OH)_2_D_3_ from the extracellular environment, VDR is activated and translocated into the nucleus where it either induce or suppress transcription of a variety of genes. As an example, VDR induce upregulation of PLC-γ1 in naïve human T cells. Once PLC-γ1 is expressed, TCR induced activation of PLC-γ1 leads to activation of PKA and PKC and an increase in the intracellular calcium level. In other cell types PKA and PKC has been shown to modulate expression of VDR, depending on the particular cell type and cellular differentiation state investigated. An increase in intracellular calcium concentration activates NFAT1 a necessary transcription factor for expression of IL-2 and other cytokines. IL-2 is a cytokine required for proliferation of T cells and one mechanism by which VDR adjust T cell activity is to outcompete NFAT1’s binding to the IL-2 promoter and furthermore to down-regulate the actual expression of NFAT1. To control VDR activity a series of negative feedback loops exists; activated VDR both induce expression of the 1,25(OH)_2_D_3_ degrading enzyme CYP24A1 and down-regulates expression of the 1,25(OH)_2_D_3_ synthesizing enzyme CYP27B1.

Only few studies have investigated whether there is coherence between VDR expression and T cell effector function. In the iNKT cell study performed by Cantorna and coworkers, a reduction of at least fifty percent in iNKT cells that produced the effector cytokine IL-4 and IFN-γ was observed in multiple organs (Yu and Cantorna, 2008). However, as iNKT cells most likely acquire the ability to transcribe IL-4 and IFN-γ during thymic development at the stage where they diverge from conventional T cells (Bezbradica et al., 2006), it is possible that the reduced cytokine production observed is due to defects in iNKT cell development. In a study of conventional T cells from VDR-KO mice, Bruce et al. (2011) showed that VDR-KO Th17 cells induced in *in vitro* cultures overproduced IL-17 as compared to WT cells. In contrast to the study performed by Cantorna using iNKT cells from VDR-KO mice, Bruce et al. found no change in IFN-γ production in the cultured conventional VDR-KO T cells. Taking this into consideration and the fact that Th17 cells are more readily induced in the VDR-KO mice, it is likely that the increased IL-17 production observed by Bruce et al. (2011) is also a developmental defect. Conversely, an *in vitro* study in human T cells performed by Youssef and coworkers favors a direct effect of VDR on IL-17 production. Here they showed that VDR blocks binding of the transcription factor NFAT1 to the promoter of the human IL-17 gene leading to a decrease in IL-17 production (Joshi et al., 2011). This inhibitory mechanism somehow resembles VDR’s control of both IL-2 and GM-CSF transcription in which VDR also inhibits NFAT1 binding to the DNA of the respective cytokine genes (Figure 1) (Alroy et al., 1995; Takeuchi et al., 1998; Towers and Freedman, 1998). As NFAT1 is a transcription factor involved in regulation of a wide range of genes and as VDR’s inhibition of NFAT1 appears not to include a canonical VDRE sequence in the promoter regions (Towers and Freedman, 1998), the transcriptional control of VDR’s target genes is likely far more widespread than first anticipated. Today, a direct effect of 1,25(OH)_2_D_3_-VDR signaling on the expression of effector T cell molecules includes not only cytokines but also chemokines and chemokine homing receptors as reviewed by Peelen et al. (2011).

Studies in which either T cell conditional VDR knock-out animals or animal models of adoptive transferred T cells from a VDR knock-out animal would substantially increase our understanding of VDR’s direct influence on T cell effector function. Along this line Hayes and coworkers developed a mouse model in which only the T cells included an inactive VDR gene in order to investigate the implication of T cells in development of autoimmunity. In this model T cells developed normally in thymus but peripheral T cells expressing an inactive VDR were resistant to the inhibitory effect of vitamin D on autoimmune disease development (Mayne et al., 2011). Future studies will likely elaborate on specific T cell effector functions in similar animal models.

### Allelic variations and non-functional VDR in the human population

Allelic variants of the VDR gene occur naturally in the human population. Even though interpretation of these polymorphic variants in relation to different diseases is difficult due to the small numbers of subjects included in the performed studies, an association with disease risk has been suggested. This includes a greater susceptibility to infections and a higher incidence of autoimmune diseases and cancer. The impact of VDR polymorphism on VDR function may in part be due to reduced VDR-mRNA stability and hence of reduced VDR expression (Feldman et al., 2005). In addition to allelic variations of the VDR gene, a rare genetic disorder has been described in which the VDR gene contain mutations that renders the gene product non-functional. This includes mutations in the DNA-binding domain and in the ligand binding domain, rendering binding of VDR to DNA, RXR, or co-regulators impossible (Malloy and Feldman, 2010). Individuals with a non-functional VDR suffer from the absence of VDR signaling giving rise to the disease hereditary vitamin D resistant rickets (HVDRR). In HVDRR patients the serum level of 1,25(OH)_2_D_3_ is exceedingly high and most patients are completely resistant to vitamin D therapy. As there are only very few cases of HVDRR, long-term effects of defective VDR signaling on immune function such as development of autoimmune diseases and control of cancer have not yet been documented (Malloy and Feldman, 2010). A promising model system regarding this issue is the VDR-KO mouse in which the VDR gene has been deleted. These mice show increased sensitivity to autoimmune diseases, and are more prone to oncogene- and chemocarcinogen-induced tumors (Bouillon et al., 2008) illustrating a possible *in vivo* relation between VDR expression and immune function.

## Regulation of VDR Expression and Activity

The studies described above have led to an understanding of the importance of VDR expression in T cell development, differentiation, and function. Even though the abundance of VDR in T cells reflects the cells responsiveness to 1,25(OH)_2_D_3_, this concept likely is far more complex. Besides transcriptional regulation of the VDR, additional factors with an impact on VDR activity should be considered. This includes ligand availability, induction of intracellular signaling pathways, posttranslational modifications of VDR, nuclear translocation, and DNA binding as well as recruitment of activated co-regulators.

### Ligand availability

The role of the VDR-ligand 1,25(OH)_2_D_3_ is to convert VDR into a functionally active protein that can bind to RXR and to specific gene sequences and co-regulators necessary for modulation of gene expression (Pike, 2011; Pike et al., 2012; Haussler et al., 2013). Availability of 1,25(OH)_2_D_3_ is therefore a prerequisite for VDR activity. The circulating concentration of 1,25(OH)_2_D_3_ is very low (≤100 pM) compared to its metabolic inactive precursor 25(OH)D_3_ (≤100 nM) (Feldman et al., 2005). During an immune reaction it is therefore most likely that the source of 1,25(OH)_2_D_3_ predominantly is endogenous production from the precursor molecule 25(OH)D_3_. In support of this, several studies of immune cells have revealed that 25(OH)D_3_ can be taken up and subsequently converted into 1,25(OH)_2_D_3_ through the action of the enzyme CYP27B1 (Figure 1) (Jeffery et al., 2012). CYP27B1 has been identified in most cells of the immune system (Fritsche et al., 2003; Liu et al., 2006; Sigmundsdottir et al., 2007; Krutzik et al., 2008; Correale et al., 2009; Baeke et al., 2010), however, it is not clear whether all cells can take up the precursor 25(OH)D_3_ and convert it. When 1,25(OH)_2_D_3_ is synthesized a great part is secreted to adjacent cells, minimizing the need for endogenous production in all immune cells (Jeffery et al., 2012). In addition, 1,25(OH)_2_D_3_ holds the capacity to restrict its own synthesis by exerting a negative feedback on the vitamin D signaling system. 1,25(OH)_2_D_3_ induces displacement of a key transcriptional factor responsible for CYP27B1 expression leading to a decrease in CYP27B1 (Murayama et al., 1999) and also induces a rapid binding of VDR-RXR to the promoter sequence of the 1,25(OH)_2_D_3_ degrading enzyme CYP24A1 leading to an increase in CYP24A1 (Figure 1) (Ohyama et al., 1996; Kim et al., 2005). The net result is a reduction in endogenous 1,25(OH)_2_D_3_. As illustrated by Vidal et al. this negative feedback mechanism can be partly prevented by inflammatory-induced proteins. In this study, they showed that IFN-γ induced activation of STAT1 promoted binding of STAT1 to the DNA-binding domain of VDR, preventing VDR from inducing expression of CYP24A1 (Vidal et al., 2002).

A major determinant of 25(OH)D_3_ availability is the carrier protein DBP that binds most circulating vitamin D in the serum. In immune reactions DBP restricts the availability of 25(OH)D_3_ to the immune cells (Chun et al., 2010; Jeffery et al., 2012). More than 100 genotypes of DBP have been documented but most people express the three most common variants GC1S, GC1F, and GC2 (Arnaud and Constans, 1993). These DBP variants have different properties including a difference in their affinity for 25(OH)D_3_ (Arnaud and Constans, 1993; Wood et al., 2011). *In vitro* studies performed with immune cells using different DBP genotypes in addition to 25(OH)D_3_ have shown that the particular genotype used influences the magnitude of the immune response (Chun et al., 2010; Jeffery et al., 2012). Along this line, an association between DBP genotype and development of inflammatory diseases has been described (Papiha and Pal, 1985; Speeckaert et al., 2006; Martineau et al., 2010). 1,25(OH)_2_D_3_ availability therefore is the sum of the circulating 25(OH)D_3_ level, DBP genotype, CYP27B1 function, near proximity to other cells that produces and secretes 1,25(OH)_2_D_3_ and 1,25(OH)_2_D_3_ self-restriction.

### Extracellular signals that modulate VDR expression

Vitamin D receptor expression can be modulated by numerous physical stimuli such as dietary composition (e.g., calcium and phosphorus), steroid hormones, growth factors, peptide hormones (Feldman et al., 2011), and inflammatory agents (Provvedini et al., 1983; Liu et al., 2006, 2009; von Essen et al., 2010; Joseph et al., 2012). For example, VDR expression is significantly regulated by the steroid hormones estrogen, glucocorticoid, and retinoids which appears to be rather cell specific (Feldman et al., 2011). The effect of glucocorticoid on VDR expression in the immune system has not been evaluated, but glucocorticoids are known to have a profound anti-inflammatory and immune suppressive effect (Miller and Ranatunga, 2012). Glucocorticoid therapy is used to suppress inflammation implicated in the pathogenesis of various inflammatory diseases (Hanaoka et al., 2012; Miller and Ranatunga, 2012), and it could be speculated that one mechanism used by glucocorticoids to suppress immune responses is by increasing the expression levels of VDR. Estrogen (Chighizola and Meroni, 2012) and retinoids (Cassani et al., 2012) also appear to have strong immunomodulatory effects, but like glucocorticoid the implication of VDR regulation as a possible mechanism to modulate immune function has not been investigated. Receptors for the peptide hormone parathyroid hormone (PTH) was recently identified on T cells (Geara et al., 2010). This renders PTH-induced modulation of VDR expression in T cells a possibility as observed for other cell types (Feldman et al., 2011). Again, this is unexplored territory even though PTH possesses an immune regulatory ability (Geara et al., 2010). The most well described hormonal effect on VDR activity and expression is that of 1,25(OH)_2_D_3_ itself, as 1,25(OH)_2_D_3_ directly influences the expression levels of VDR by homologous regulation. Although varying between different cell types, 1,25(OH)_2_D_3_ in general increases VDR-mRNA production (McDonnell et al., 1987), stabilizes VDR-mRNA, and protects the VDR against degradation (Feldman et al., 2005), altogether increasing the total amount of the VDR.

Various inflammatory signals have also been shown to induce upregulation of VDR in immune cells. During an innate immune response, pathogen-induce activation of toll-like-receptors on human monocytes and macrophages results in upregulation of the VDR (Liu et al., 2006). Likewise, antigen-induced activation of TCR on human naïve T cells induce upregulation of the VDR (Provvedini et al., 1983; von Essen et al., 2010; Joseph et al., 2012). Furthermore, T cell cytokines induced during inflammation can modulate VDR expression (Edfeldt et al., 2010; Spanier et al., 2012), illustrating that regulation of the VDR level is a common mechanism used in the defense against pathogens.

### Intracellular signaling pathways that modulate VDR expression

Modulation of VDR expression as a result of physical stimuli is mediated by various intracellular signaling pathways. Although only a sparse numbers of publications concern this issue, a few studies agree that activation of the cAMP-dependent protein kinase A (PKA) pathway leads to an increase in VDR abundance (Pols et al., 1988; Krishnan and Feldman, 1992; Song, 1996). Both cellular responses to PTH (Pols et al., 1988) and to prostaglandin (Smith et al., 1999) activate PKA causing an increase in the VDR level. In contrast, Feldman and coworkers showed that stimuli that induce protein kinase C (PKC) activity down-regulate both VDR-mRNA and VDR protein levels in fibroblastic cells (Krishnan and Feldman, 1991). Moreover, Reinhardt and Horst (1994) has shown that the impact of PKC activation on the VDR-mRNA level highly depends on the particular cellular differentiation state investigated. This suggests that other signaling pathways may cooperate to determine the final effect on VDR expression. In support of this idea, a study by Krishnan and Feldman (1992) indicated a mutual antagonism between the PKA and PKC pathways in regulation of the VDR level, an observation confirmed by others (van Leeuwen et al., 1992). Furthermore, it has been suggested that the intracellular calcium level that is known to influence and be influenced by PKC activity is implicated in PKA induced VDR upregulation (Figure 1) (van Leeuwen et al., 1990). A new signaling pathway which leads to VDR expression has recently been described in human naïve T cells. Here, TCR stimulation induces VDR expression through activation of the p38 mitogen activated protein kinase by ZAP-70 (Figure 1) (von Essen et al., 2010). In contrast, Gocek et al. (2007) showed that VDR expression was controlled by Erk and PI3K signaling in a myeloid leukemia cell line where p38 activity appeared irrelevant. This implies that not only might different intracellular signaling pathways cooperate to regulate the expression of VDR, but also that the implicated signaling events differs between different cell types and different differentiation states of the cells.

### Transcriptional regulation of VDR

Until recently the regulatory responses to hormones at the VDR-gene promoter were unknown. To clarify this, Zella et al. (2010) used ChIP–chip analysis to investigate the VDR gene transcription. These investigations revealed the presence of several enhancers, including the transcription factor C/EBPβ involved in basal expression of VDR as well as the transcription factor glucocorticoid receptor (GR) which mediates the action of glucocorticoids, the transcription factor retinoid acid receptor (RAR) mediating the action of retinoic acid, and the transcription factor CREB mediating the action of PTH (Zella et al., 2010). In case of VDR enhancement by 1,25(OH)_2_D_3_, Zella et al. (2006, 2010) found accumulation of VDR-RXR and RNA pol II at the VDR gene together with an increase in C/EBPβ binding. They also detected a substantial increase in histon H4 acetylation associated with enhancer regions across the VDR locus (Zella et al., 2010). An induction of transcription from promoters is often associated with an increase in H4 acetylation, and the observations therefore indicated the existence of multiple enhancers in the VDR-gene locus that may contribute to 1,25(OH)_2_D_3_-induced VDR expression. Transcriptional regulation of the VDR gene therefore includes the presence and activity of a wide range of enhancers induced by extracellular signals as well as induction of various epigenetic changes. In case of inflammatory-induced VDR upregulation, the regulatory responses at transcriptional level have not been investigated. As new techniques such as ChIP–chip and ChIP–seq have emerged, this topic will likely be explored in nearby future.

### Posttranslational modifications of VDR

In addition to transcriptional regulation of VDR, several *in vitro* studies have suggested that VDR can be post-translationally modified. Studies by Haussler and coworkers revealed that 1,25(OH)_2_D_3_ binding to VDR led to serine phosphorylation at multiple sites of the receptor. PKC was implicated in phosphorylation at serine 51, an event that partly inhibited VDR transcriptional activity (Hsieh et al., 1991). Although not required for VDR transcriptional activity, casein kinase II (CK II)-induced phosphorylation at serine 208 led to an enhancement of VDR transcriptional activity (Jurutka et al., 1996). As both PKC and CKII activity is induced in cells in response to various stimuli, it can be proposed that these posttranslational modifications although probably not obligatory for VDR function represents a mode to adjust the activity of VDR according to the specific signals received by the cell. Disease-induced posttranslational modifications leading to a dysfunctional VDR has also been documented. In a study by Patel et al. (1995) plasma toxins from uremic patients was shown to bind to the patients VDR, thereby disrupting binding of VDR-RXR to DNA resulting in a diminished VDR response. It so appears that posttranslational modifications of VDR adjust VDR activity in both health and disease.

### RXR and other co-regulators of VDR

The genomic actions of 1,25(OH)_2_D_3_ also highly depends on the abundance and activity of proteins that interact with VDR. Binding of VDR to its ligand 1,25(OH)_2_D_3_ facilitates association with RXR and in the absence of RXR, VDR is unable to bind to most VDRE in vitamin D target genes (Kliewer et al., 1992; Forman et al., 1995; Chambon, 1996). In addition to RXR binding, VDR interacts with various co-activators or co-repressors once bound to the DNA (Nagpal et al., 2005; Pike et al., 2012; Haussler et al., 2013). These co-regulatory complexes are necessary for the VDR-RXR heterodimer to either induce or suppress gene transcription and include ATPase-containing nucleosomal remodeling capabilities, enzymes with chromatin histone modifying abilities (e.g., acetyl- or methyl-transferases), and proteins involved in recruitment of RNA polymerase II (Pike et al., 2012). GRIP1 (Issa et al., 2001), RAC3 (Issa et al., 2001), SRC-1 (Masuyama et al., 1997a), TIF-1 (vom et al., 1996), ACTR (Chen et al., 1997), pCIP (Torchia et al., 1997), and Mediator (Oda et al., 2010) are some of the described co-activator proteins and co-activator complexes to date. These co-activators all regulate VDR function through co-assembling with VDR but they modulate VDR activity via distinct mechanisms. GRIP1 and RAC3 for example regulate VDR activity by modulating crosstalk between VDR and RXR (Issa et al., 2001), ACTR encompass histone acetyltransferase capacity and can recruit other nuclear factors (Chen et al., 1997), and Mediator which is a large complex composed of several MED-proteins activates transcription by direct recruitment of the RNA polymerase II transcriptional machinery (Oda et al., 2010). Although most co-activators facilitate VDR-induced transcriptional activation by binding to VDR, others are shown to be released from VDR to enable transcription, e.g., TFIIB (Masuyama et al., 1997b); illustrating the functional complexity of these co-activator complexes. Only a few co-repressor proteins involved in VDR silencing of genes have been described. As an example NcoR-1, NcoR-2, and Hairless can recruit histone deacetylase activity to VDR-target genes, leading to chromatin compaction and hence gene silencing (Nagpal et al., 2005). A recent study by Singh et al. (2012) furthermore showed that recruitment of co-repressors inappropriately can change during disease, causing a deregulation of VDR-target genes. In addition to transcriptional control of VDR, co-regulator proteins can modulate VDR abundance by enhancing degradation of VDR. Certain cellular signaling events have been shown to motivate the physical interaction of VDR-1,25(OH)_2_D_3_ with SUG1 of the proteasome complex, targeting VDR for ubiquitination and subsequent proteolysis (Masuyama and MacDonald, 1998). Therefore, it is evident that regulation of the expression level of RXR and other co-regulators are important to modulate the activity of VDR, and it could be speculated that expression of particular co-regulators are dictated by the inflammatory environment.

### T cells modulate VDR expression in other immune cells

A recent study by Edfeldt et al. revealed that VDR expression is not only modulated on a single cell level. Their study showed that VDR expression of innate immune cells could be regulated by nearby T cells (Edfeldt et al., 2010). In innate immunity, pathogen-induced signaling through Toll-like-receptors on human monocytes and macrophages up-regulate the expression of VDR. This in turn, leads to VDR-induced expression of the antimicrobial peptide cathelicidin resulting in killing of microbes (Liu et al., 2006). VDR-induced cathelicidin expression by human monocytes was shown to be adjusted by cytokines produced by T cells. By modulating the level of VDR and the amount of VDR-ligand available by adjusting the CYP27B1 level, the T cell cytokine IFN-γ increases cathelicidin expression and IL-4 attenuates cathelicidin expression (Edfeldt et al., 2010). This example illustrates how interplay between innate and adaptive immunity cooperates to mount an appropriate response to infection through regulation of the VDR-system.

## Concluding Remarks

This review indicates that VDR expression and activity are important for all stages of a T cells life, ranging from development to differentiation and elicitation of effector functions. In concordance, VDR expression and activity are associated with immunity against certain infections and with the prevalence of some autoimmune diseases. In animal models 1,25(OH)_2_D_3_ has been shown to prevent development of autoimmune diseases. This includes experimental autoimmune encephalomyelitis (EAE), the animal model for MS (Mayne et al., 2011). EAE studies performed in VDR-KO animals (Bouillon et al., 2008) or in animals with a dysfunctional VDR (Mayne et al., 2011) illustrates the requirement of a functional VDR in 1,25(OH)_2_D_3_ mediated EAE-inhibition. Furthermore, a study by Hayes and coworkers showed that VDR-gene inactivation selectively in the T cells completely eradicated the ability of 1,25(OH)_2_D_3_ to inhibit EAE (Mayne et al., 2011). The biological relevance of low levels of VDR in development of MS was confirmed in a microarray analysis performed by Achiron et al. Here they compared blood mononuclear cells from healthy subjects that later developed MS with healthy subjects that remained MS-free. One of the early disease markers identified turned out to be suppressed VDR expression (Achiron et al., 2010). These observations may not only reflect a change in conventional T cells (e.g., development of more memory T cells that are predisposed to develop into Th1 and Th17 cells as observed in VDR-KO mice (Bruce et al., 2011) but also a reduced development of iNKT cells (as observed in VDR-KO mice, Yu and Cantorna, 2008; Ooi et al., 2012). iNKT cells are negative regulators of EAE (Matsuda et al., 2008) and furthermore, fewer iNKT cells can be found in the blood of MS patients (Araki et al., 2003). Along this line Araki et al. (2003) showed that an increase in iNKT cell number is associated with remission from symptoms in MS patients. Altogether, these observations emphasize a role for VDR expression in development and progression of autoimmunity.

Most experiments investigating susceptibility to a given autoimmune disease is, however, based on animal models. The question therefore remains whether these animal models which are executed in a pathogen free environment reflect the real life situation where humans continuously are bombarded with a variety of pathogens. It is slowly becoming apparent that the microbial environment has a greater influence on development of autoimmune diseases than previously anticipated. For example, certain microbes have been shown to slow innate immune defenses by dysregulating the VDR. One mechanism used by the innate immune system to clear a pathogen is VDR-induced production of the antimicrobial peptide cathelicidin which possesses antiviral, antibacterial, and antifungal activity. Therefore, any microbe capable of dysregulating expression of the VDR would enhance its chance for survival (Waterhouse et al., 2009; Proal et al., 2013). Klein and coworkers illustrated *in vitro* that Epstein-Barr virus (EBV) were able to effectively down-regulate expression of VDR in B cells (Yenamandra et al., 2009), Modlin and coworkers that *Mycobacterium leprae* inhibits VDR activity through down-regulation of CYP27B1 in monocytes (Liu et al., 2012), Wang and coworkers that *Mycobacterium tuberculosis* down-regulate expression of VDR in macrophages (Xu et al., 2003), and McElvaney and coworkers that the fungus *Aspergillus fumigates* secretes a toxin capable of down-regulating VDR in macrophages (Coughlan et al., 2012). This allows pathogens to accumulate in tissue and blood and the weakened innate defense further causes susceptibility to additional infections. As more pathogens are incorporated into this microbiome, people start to show symptoms characteristic of inflammatory and autoimmune diseases. Accumulating evidence now supports the observation that a number of autoimmune diseases can be reversed by restoring VDR function (using the VDR agonist olmesartan) along with antibiotics. This includes rheumatoid arthritis, systemic lupus erythematosis, sarcoidosis, scleroderma, psoriasis, Sjogren’s syndrome, autoimmune thyroid disease, and type I and II diabetes mellitus (Waterhouse et al., 2009; Proal et al., 2013). Knowledge of the regulation of VDR abundance and activity in immune cells potentially is of great therapeutic importance, and therapeutic enhancement of VDR should therefore be considered in the clinic today.

## Conflict of Interest Statement

The authors declare that the research was conducted in the absence of any commercial or financial relationships that could be construed as a potential conflict of interest.
